# Methylseleninic Acid Provided at Nutritional Selenium Levels Inhibits Angiogenesis by Down-regulating Integrin β3 Signaling

**DOI:** 10.1038/s41598-017-09568-5

**Published:** 2017-08-25

**Authors:** Zhihui Cai, Liangbo Dong, Chengwei Song, Yanqing Zhang, Chenghui Zhu, Yibo Zhang, Qinjie Ling, Peter R. Hoffmann, Jun Li, Zhi Huang, Wei Li

**Affiliations:** 1 Department of Biotechnology, Jinan University, Guangzhou, Guangdong Province China; 2 College of Pharmacy, Jinan University, Guangzhou, Guangdong Province China; 3 Department of Cell and Molecular Biology, John A. Burns School of Medicine, University of Hawaii, Honolulu, Hawaii USA

## Abstract

Targeting angiogenesis has emerged as a promising strategy for cancer treatment. Methylseleninic acid (MSA) is a metabolite of selenium (Se) in animal cells that exhibits anti-oxidative and anti-cancer activities at levels exceeding Se nutritional requirements. However, it remains unclear whether MSA exerts its effects on cancer prevention by influencing angiogenesis within Se nutritional levels. Herein, we demonstrate that MSA inhibited angiogenesis at 2 µM, which falls in the range of moderate Se nutritional status. We found that MSA treatments at 2 µM increased cell adherence, while inhibiting cell migration and tube formation of HUVECs *in vitro*. Moreover, MSA effectively inhibited the sprouts of mouse aortic rings and neoangiogenesis in chick embryo chorioallantoic membrane. We also found that MSA down-regulated integrin β3 at the levels of mRNA and protein, and disrupted clustering of integrin β3 on the cell surface. Additionally, results showed that MSA inhibited the phosphorylation of AKT, IκBα, and NFκB. Overall, our results suggest that exogenous MSA inhibited angiogenesis at nutritional Se levels not only by down-regulating the expression of integrin β3 but also by disorganizing the clustering of integrin β3, which further inhibited the phosphorylation involving AKT, IκBα, NFκB. These findings provide novel mechanistic insight into the function of MSA for regulating angiogenesis and suggest that MSA could be a potential candidate or adjuvant for anti-tumor therapy in clinical settings.

## Introduction

Angiogenesis is a process of forming new blood vessels from existing vasculature that is strictly regulated by multiple factors and is vital to growth and development^1–3^. However, mounting evidence suggest that imbalance of angiogenesis triggers the progressions of many diseases including malignant tumors^4, 5^, supporting investigations into angiogenesis inhibitors for cancer treatment. Angiogenesis initially involves cell proliferation and migration^4^, which is induced by secretion of growth factors such as vascular endothelial growth factor (VEGF)^2^. VEGF exerts its effects by activating multiple cellular events within the vascular endothelium including cell proliferation, migration, and tube formation^6, 7^. Additionally, angiogenesis is also regulated by integrin family members that modulate cell polarity, junction, proliferation, migration and signal transduction^8–10^. The integrin family has been found to have more than two dozen unique heterodimers formed by the combination of eighteen α and eight β subunits^8^. Among these integrin subunits, integrin β3 has been found to be highly expressed in endothelial cells. During angiogenesis, conformational changes and clustering states of integrin β3 initiate intracellular signal transduction through the phosphorylation cascade of FAK, AKT, IKKα, IκBα, and NF-κB^11, 12^. Thus, integrin β3 signaling regulates the downstream expression of cytokines (TNFα, IL-1β, IL-6), adhesion molecules (VCAM-1, ICAM-1, E-selectin) and enzymes (iNOS, COX-2) during angiogenesis^13–16^. Moreover, integrin β3 has a concentrated cell surface distribution at the leading edge in motile cells that regulates cell polarity and directs migration^17, 18^.

Selenium (Se) is an essential micronutrient for mammals that exhibits antioxidant and anti-cancer potential for human health. Se metabolites and compounds include methylseleninic acid (MSA), selenodiglutathione (SDG), and hydrogen selenide (H_2_Se), which have been reported to exhibit chemopreventive effects against different types of cancer^19^. However, the narrow window between their effective and toxic dosages limit its use and highlight the importance of determining effective dosages and forms of Se metabolites and compounds^20, 21^.The recommended dietary allowance (RDA) of Se supplementation in the U.S. is 55 μg/day for adults^22^. Many publications have reported the Se nutritional status in whole blood ranging from 0.71 to 3.24 μM of subjects with moderate Se status. There is good correlation between blood Se concentration and the activity of the selenoenzyme, glutathione peroxidase (GPx), in the plasma and most cells. Therefore, the Se content of physiological fluids may be considered a reliable index of status with respect to nutritional Se^23^.

Recent studies have focused on organic forms of dietary Se, which show higher bioactivity but lower cytotoxicity. MSA is an organic species of Se that is metabolized from animal cells^20^. It exhibits anti-oxidative and anti-cancer activity at level above moderate Se nutritional concentrations. Unique features of MSA that all contribute to its anti-cancer activities include its capacity to induce ROS accumulation, to inhibit the phosphorylation of AKT, ERK and p53, and to facilitate proteolytic activation of caspase-8 and -3^20, 24–26^.Moreover, metabolism of MSA generates the precursor of methylselenol, which is an active Se form and reported to induce the apoptosis of cancer cells^27^. However, little information is known about the effects of MSA in angiogenesis within the physiological range of nutritional Se intake, and it remains unclear whether MSA influences angiogenesis related to integrin β3 signaling.

For the current study, we utilized human umbilical vein endothelial cells (HUVECs) as an *in vitro* model and focused on molecular mechanisms by which MSA inhibited angiogenesis. Our results showed that exogenous MSA provided nutritional Se levels effectively inhibited cell migration, tube formation, and neoangiogenesis by down-regulating integrin β3 and interrupting the clustering of integrin β3. Additionally, MSA inhibited the phosphorylation of AKT, IκBα, and NF-κB, thereby highlighting the anti-angiogenic capacity of MSA.

## Results

### Uptake and metabolism of MSA by HUVECs

**Figure 1:**
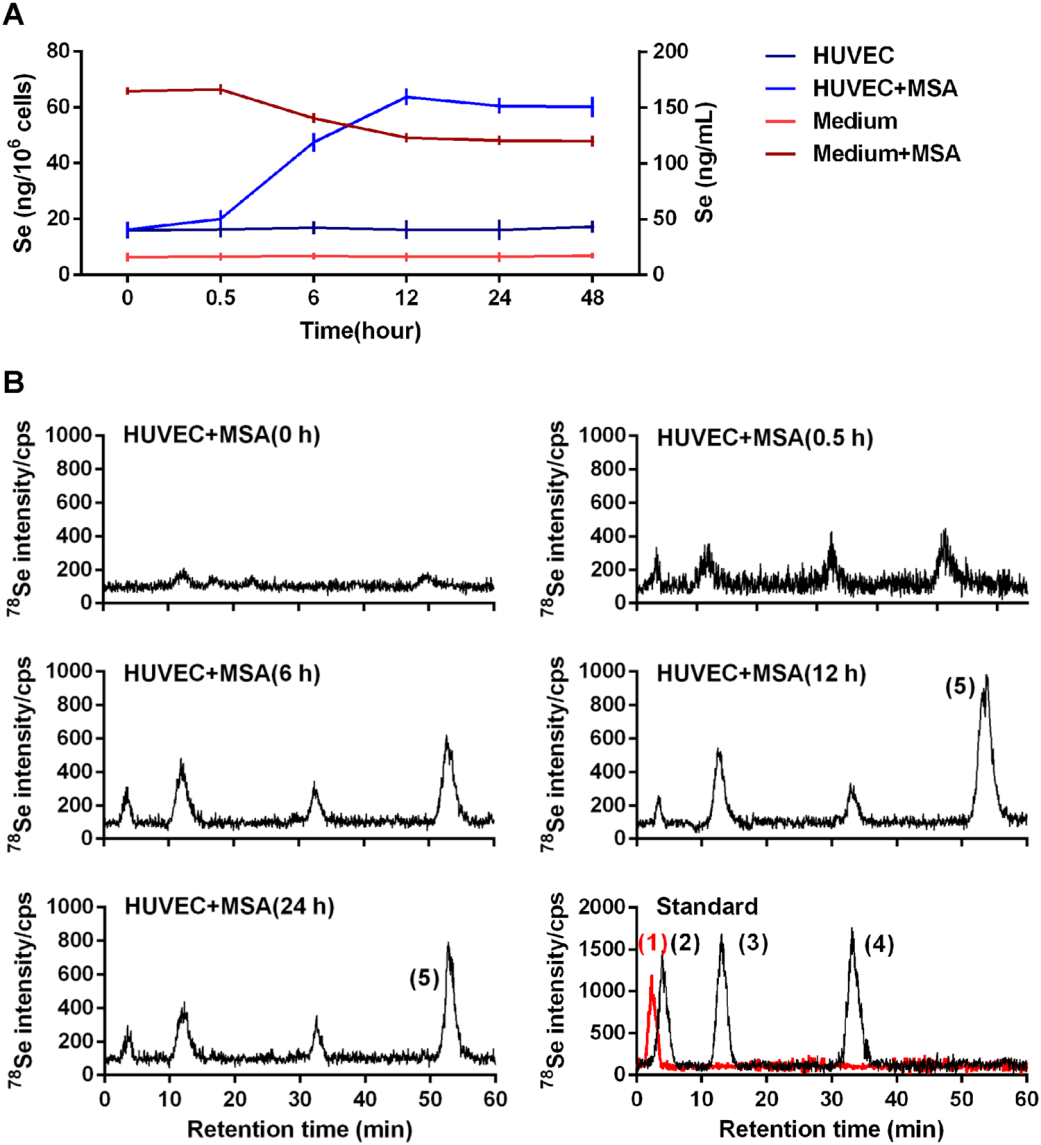
Uptake and metabolism of MSA by HUVECs. (**A**) Se concentrations in medium or cells treated with/without 2 μM MSA for different hours (0, 0.5, 6, 12, 24, 48 h) were detected by AFS. The *error bars* represented the SD (n = 3). (**B**) Se metabolites of HUVECs exposed to 2 μM MSA for different hours (0, 0.5, 6, 12, 24 h) were detected by HPLC-ICP-MS, and different selenium standards including MSA, SeMet, SeMC, and DMSe were used as positive control. Peak identification: (1) MSA, (2) SeMC, (3) SeMet, (4) DMSe, (5) Unknown.

In order to monitor the dynamic uptake and metabolism of MSA when provided at nutritional Se concentrations, HUVECs were treated with 2 μM MSA for 0 to 48 h. Se concentrations in cell lysates and culture medium were analyzed by Atomic Fluorescence Spectroscopy (AFS). As shown in Fig. 1A, Se accumulated in HUVECs with this range of MSA exposure, and reached maximum levels of 63.75 ng/10^6^ cells at a longer exposure time (12 h). There was a trend towards slightly decreased but still maintained at a higher level in intracellular Se concentration within 48 h exposure. In contrast to the changes of intracellular Se, the concentration of Se in medium was relatively stable over the first 6 h, and then it gradually decreased following the uptake by HUVECs for 48 h incubation. To quantify the endogenous Se levels, total Se and free Se (separated by ultrafiltration with 1 kDa cut-off) were detected in FBS, medium, cultured supernatant and HUVECs, in which total Se were 73.4 ± 9.6 ng/ml, 14.9 ± 2.0 ng/ml, 8.9 ± 1.6 ng/ml and 16. 8 ± 1.4 ng/10^6^ cells including free Se were 23.8 ± 4.9 ng/ml, 7.8 ± 0.8 ng/ml, 3.8 ± 2.6 ng/ml and 3.7 ± 0.7 ng/10^6^ cells, respectively (Supplementary Fig. S1A,B).

The presence of minor methyl-Se metabolites at low levels (10 μg/kg Se as SeMet or SeMC) has been reported for the lymphoma cells exposed to MSA using speciation analysis of cell lysates by reversed-phase HPLC-APEX-Q-ICPMS^28^. HPLC-ICP-MS chromatograms of 1:1 diluted lysates of HUVECs exposed 2 μM MSA for 0 to 24 h are shown in Fig. 1B. The results indicated that the Se metabolites with retention time similar to that of SeMC (tR = 4 min), SeMet (tR = 12.5 min) and DMSe (tR = 34 min) were the major Se species in HUVEC after exposure to MSA. The separated retention curve of MSA standard (200 μM) was shown in Fig. 1B, in which the retention time of MSA was approximately 2.5 min. It should be noticed that the peak of MSA (2 μM) was failed to be detected in the present experiment conditions. Meanwhile, the endogenous MSA also could not be identified and detected in FBS, medium and/or cell cultures (data not shown). Together, results showed that HUVECs could uptake and transformed exogenous MSA to other Se species within 6 h, during which cellular Se reached to the maximum level and maintained such high level within 48 hours.

### Effects of MSA on cell proliferation of HUVECs

**Figure 2:**
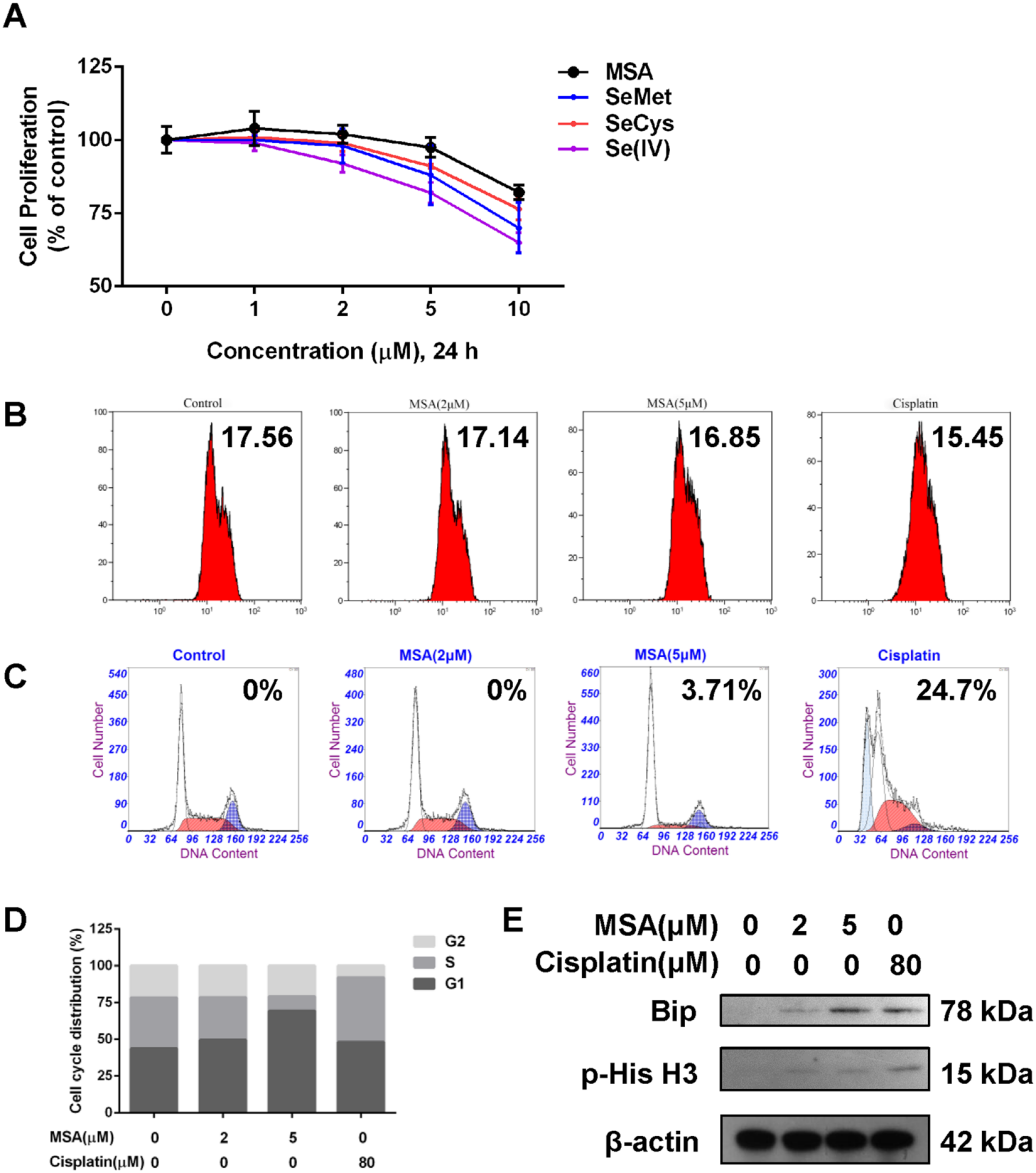
Effects of MSA on viability. (**A**) The effect of four selenium compounds including SeMet, SeCys, Se(IV), and MSA on HUVEC proliferation were detected using MTT assay. The *error bars* represented the SD (n = 3). The effect of MSA (0, 2, 5 μM, 24 h) on (**B**) mitochondrial membrane potential, (**C**,**D**) cell cycle distribution, and (**E**) protein levels for p-His and Bip were detected individually. Cisplatin was used as positive control and β-actin was served as loading control.

**Figure 3:**
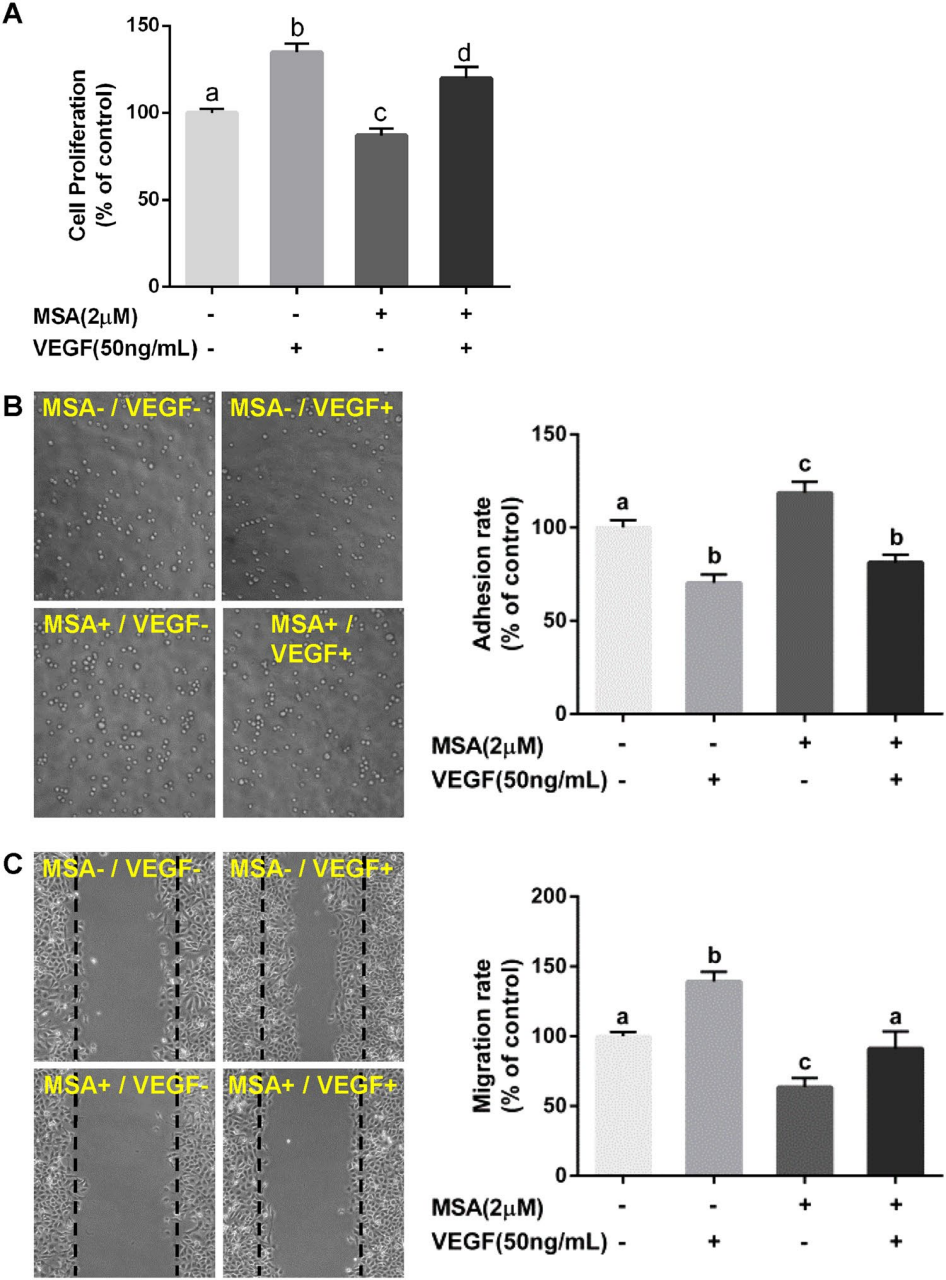
MSA increases cell adhesion but inhibits cell migration. HUVECs were treated with MSA (2 μM) and/or VEGF (50 ng/mL) for 24 h. (**A**) Cell proliferation and (**B**) numbers of adherent cells were quantified by MTT assay. (**C**) The healing distances were measured by Photoshop CS6 software. The data in Fig. 3 were determined by one way ANOVA comparison test and the *error bars* represented the SD (n = 3). Different letters indicate statistically significant differences between groups (*p* < 0.05).

First, we examined the effects of different Se compounds on cell proliferation in HUVECs. Four different Se compounds (selenocysteine, selenomethionine, sodium selenite, and MSA) at doses of 0–10 µM were tested. Results showed no effect on cell proliferation at low doses (1–2 µM), while the cells treated with MSA consistently exhibited the highest levels of cell proliferation compared with other Se compounds at higher dosage (Fig. 2A). Even so, MSA still had an inhibitory effect on cell proliferation to HUVECs at 10 µM (82.1%). Moreover, we also found that 2 μM MSA had no significant effect on VEGF-induced cell proliferation (Fig. 3A).

To further analyze the effect of MSA on apoptosis at 2 µM, Rhodamine-123 staining and PI staining were employed individually. Compared with the positive control (cisplatin, 80 µM), HUVECs exposed to 2 µM MSA showed a much higher mitochondrial membrane potential (17.14, Fig. 2B). In addition, even though no apoptotic cells were detected (Fig. 2C), exposure to 2 µM MSA could still induce a minor G1/S arrest (49.3%, Fig. 2D).

These findings were also supported by analyses of Bip or phosphorylated His on protein levels, which represent markers of protein or DNA damage during apoptosis. As shown in Fig. 2E, relatively minor changes of Bip (0.36) and p-His (0.22) were observed in the MSA-treated groups (2 µM) versus positive control (Fig. 2E). Together, these results suggest that treating with 2 µM MSA would not induce apoptosis in HUVECs.

### MSA effectively increases the adherence to collagen I and inhibits cell migration of HUVECs

Adherence and migration are crucially regulated events for tube formation in angiogenesis. Thus, we firstly examined the adhesion of HUVECs to rat-tail collagen I using an adhesion assay. As shown in Fig. 3B, MSA-treated group showed increased adherent cells (118.53%) compared with untreated controls. Next, we performed wound healing assays to examine the effects of MSA on cell migration. As shown in Fig. 3C, cells in MSA-treated group (78.28%) showed a lower migration rate compared with untreated controls, and MSA could also inhibit VEGF-induced migration (90.97% versus 139.12%). In addition, no obvious changes of cell adhesion and migration on HUVECs were detected with treatments of the endogenous Se (Supplementary Fig. S2A,B). Taken together, our results demonstrate that MSA treatment increases the adherence of HUVECs to collagen I and reduces the HUVEC migration.

### MSA down-regulates Integrin β3 and inhibits phosphorylation of AKT

**Figure 4:**
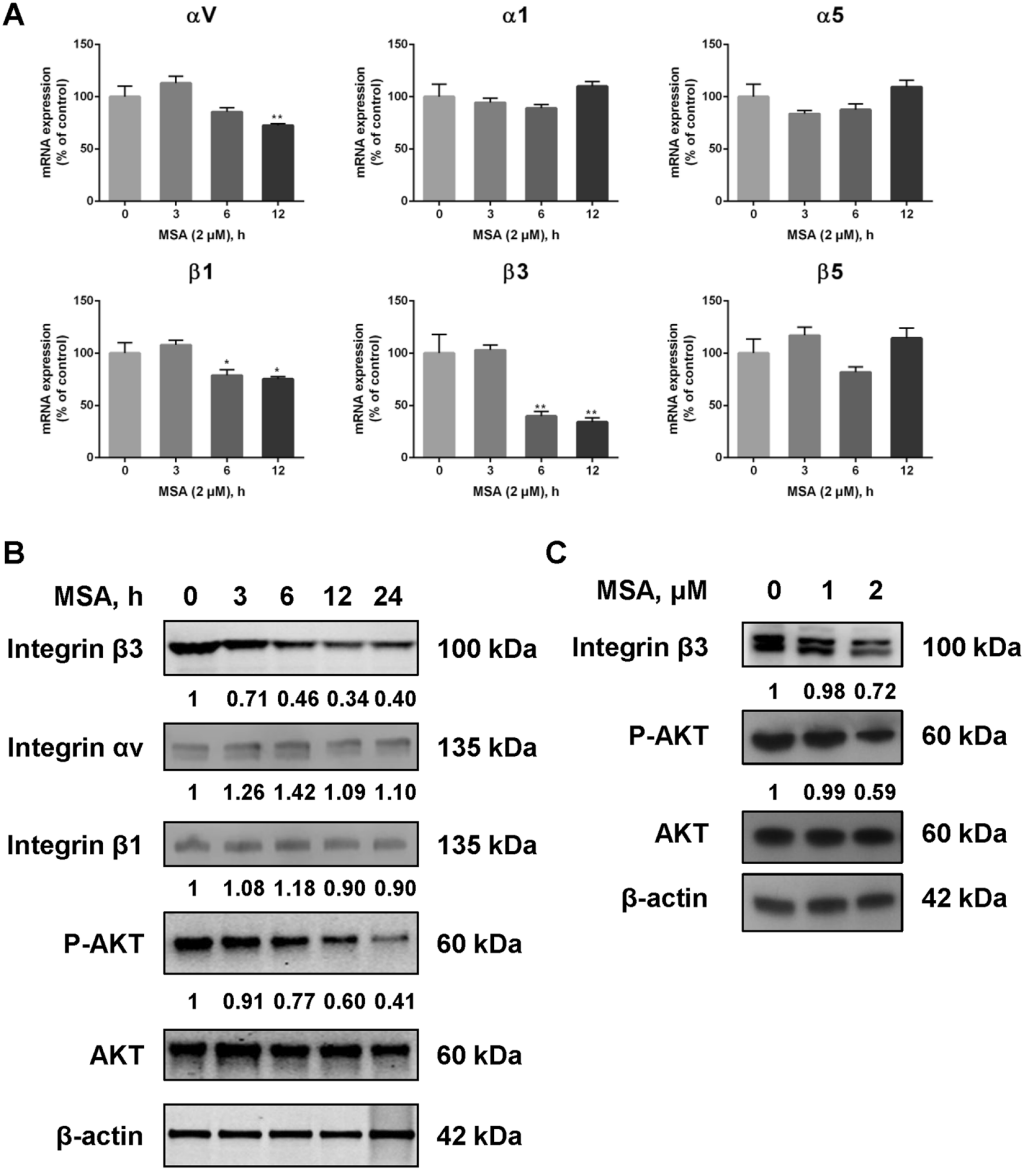
MSA down-regulates integrin β3 and inhibits phosphorylation of AKT. **(A)** Relative mRNA expressions of integrin subunits (αV, α1, α5, β1, β3, β5) in HUVECs exposed to 2 μM MSA for different hours (0, 3, 6, 12 h) were quantified by qPCR assay. A 2^−ΔΔCT^ relative quantification method was employed and β-actin was used as housekeeping gene. The *error bars* represented the SD (n = 9) and were determined by one way ANOVA comparison test. *p < 0.05, **p < 0.01. Western blot was employed to analyze the protein levels of integrin αv, β1, β3 and AKT phosphorylation in (**B**) a time-dependent (2 μM, 0, 3, 6, 12, 24 h) or (**C**) a dose dependent (0, 1, 2 μM, 24 h) manner of MSA, and β-actin was served as loading control. The relative levels of protein were quantified by ImageJ software.

Because integrins play an important role in cell adhesion, migration and signal transduction, we explored the potential involvement of integrins in MSA exposure. The mRNA levels of integrin subunits including αv, α1, α5, β1, β3 and β5 are expressed at relatively higher level in HUVECs, which were analyzed via qPCR as candidate targets of MSA. Results showed that MSA treatments only caused a significant downregulation of integrin β3 within 12 hours, while no significant changes of α1, α5 and β5 were observed (Fig. 4A). Western blot was further employed to evaluate the effect of MSA on αv, β1, and β3 on protein levels. Down-regulation of integrin β3 was observed in a time- and dose-dependent manner (Fig. 4B and C), while no significant changes of αv and β1 were observed (Fig. 4B). As a critical downstream mediator of integrins, AKT and its phosphorylation were also evaluated. Results showed that MSA also inhibited AKT phosphorylation in a time- and dose-dependent manner (Fig. 4B and C).

### MSA disrupts the clustering of integrin β3 surface localization

**Figure 5:**
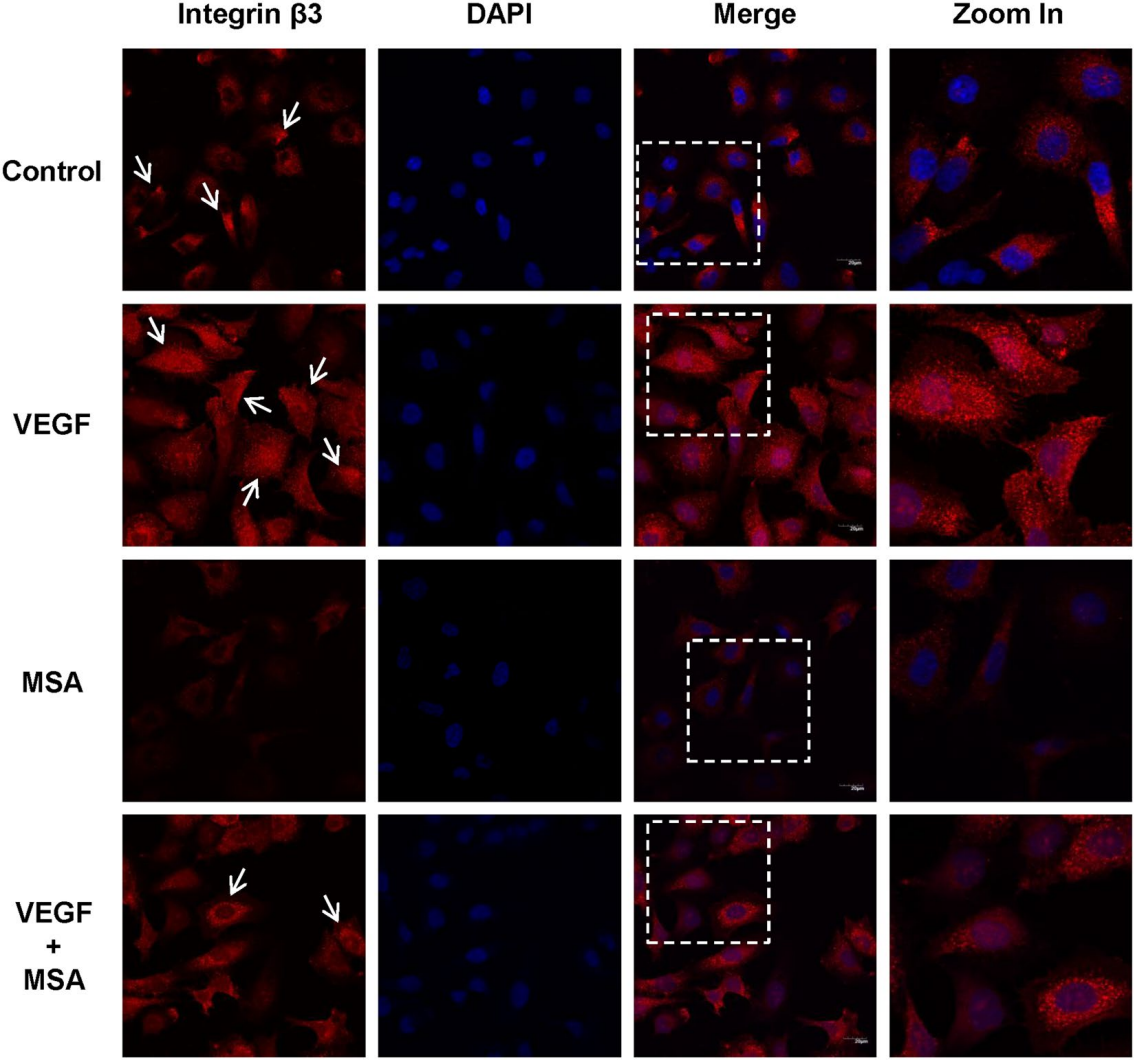
MSA disrupts the distribution of integrin β3 on cell membranes. HUVECs were treated with MSA (2 μM) and/or VEGF (50 ng/mL) for 12 h before fixation. Integrin β3 was detected using mouse monoclonal antibody against integrin β3 followed by Cy3-labelled goat anti-mouse-IgG (red) antibodies. Nuclei were counterstained with DAPI (blue). Photos were taken by confocal laser scanning microscope (FV10i).

Integrin β3 shows a clustering and polar distribution in motile cells. To determine whether MSA would interfere this effect, immunofluorescence was employed. As shown in Fig. 5, although polar distribution could not be observed, fluorescent spots (arrowheads) were fully distributed in cells exposed to VEGF. However, the fluorescence intensity and spots were significantly decreased in cells exposed to MSA compared with untreated control group, as well as in cells co-treated with MSA versus VEGF-treated group. Altogether, these data indicated that MSA treatment not only down-regulates the expression of integrin β3 but also disrupts its clustering on membrane, which may further influence the migration of HUVECs during angiogenesis.

### MSA inhibits VEGF-induced angiogenesis *in vitro*, *ex vivo* and *in vivo*

**Figure 6:**
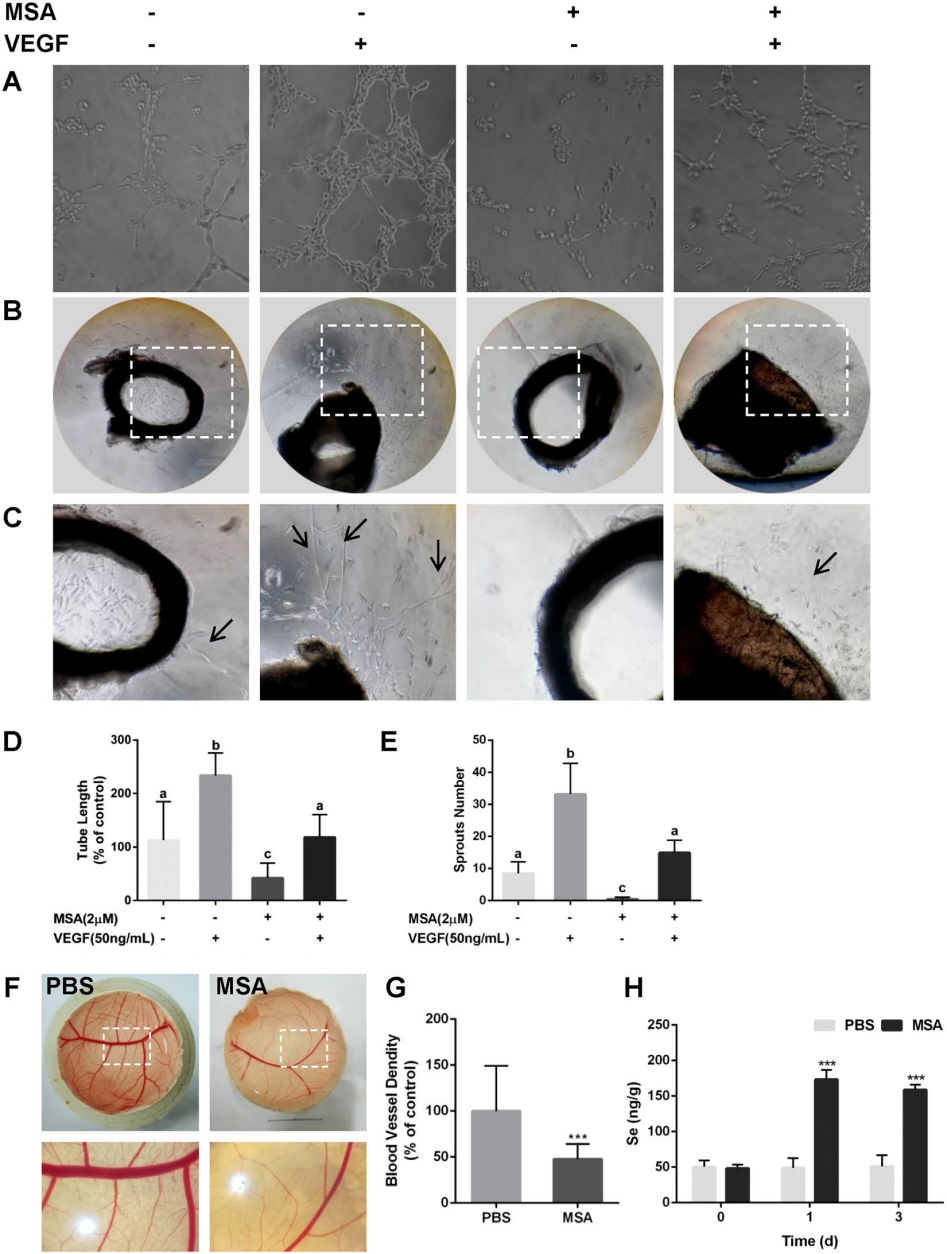
MSA inhibits VEGF-induced angiogenesis *in vitro, ex vivo* and *in vivo*. HUVECs or aortic rings were treated with MSA (2 μM) and/or VEGF (50 ng/mL). (**A**,**D**) The lengths of tubular structure were measured by Photoshop CS6 software. **(B**,**C** and **E**) The numbers of sprouts from aortic rings were counted. The *error bars* (D–E) represented the SD (D: n = 3; E: n = 60) and were determined by one-way ANOVA comparison test. Different letters indicate statistically significant differences between groups (*p* < 0.05). The blood vessels density (**F**,**G**) on CAMs were quantified by ImageJ software and the changes of Se concentrations (**H**) in CAMS treated with/without 2 μM MSA were detected by AFS. The *error bars* represented the SD (n = 6, ***p < 0.001) and were determined by nonparametric tests (**G**) or two way ANOVA comparison test (**H**).

Although angiogenesis is a complex process, tube formation is one of the crucial steps. As MSA could inhibit cell migration and down-regulate integrin β3, we further evaluated the effect of MSA on angiogenesis *in vitro* through tube formation assays. As shown in Fig. 6A and D, fewer tubular structures and elongated cells were observed in MSA-treated group (40.93%) compared with vehicle control. Although HUVECs became elongated and formed obvious tubular structures in the presence of VEGF (233.34%), these effects could be inhibited by co-treating with MSA (107.04%).

To further evaluate the anti-angiogenic effect of MSA, *ex vivo* aortic ring assays as well as *in vivo* CAM assays were performed. As shown in Fig. 6B,C and E, no microvessel sprouts were observed around aortic rings exposed to MSA compared with vehicle control, but the microvessel sprouts (arrowheads) induced by VEGF were obviously observed, which could be inhibited by co-treating with MSA. Furthermore, blood vessels density (47.81%) was significantly reduced on CAMs exposed to MSA compared with PBS-treated group (Fig. 6F and G). Total Se accumulation in CAMs reached to 173.17 ng/g after 24 h exposure to MSA at 2 µM, and a ~9% decrease within 3 days indicated a relatively high Se status in the treated CAMs. In addition, the endogenous Se could not induced obvious effects on angiogenesis (Supplementary Figs S2 and S3) under the present experimental conditions. Taken together, these results suggested that exogenous MSA supplementation was capable of suppressing angiogenesis *in vitro*, *ex vivo*, and *in vivo*.

### MSA inhibits the phosphorylation of IκBα and NF-κB, and the nuclear translocation of NF-κB

**Figure 7:**
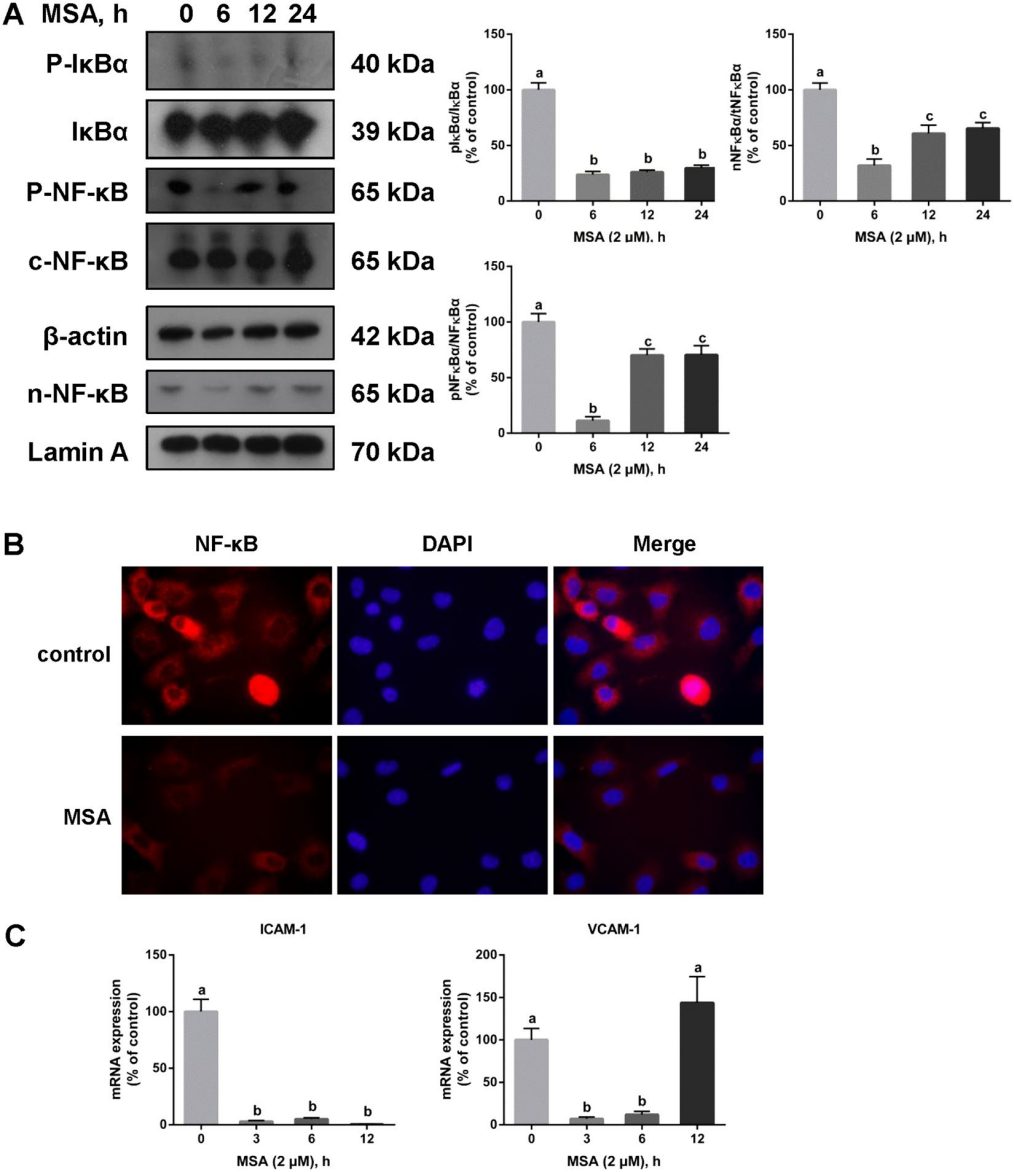
MSA inhibits the phosphorylation and nuclear translocation of NF-κB. (**A**) Western blot analyses of the effects of MSA (2 μM, 0, 3, 6, 12, 24 h) on p-IκB, p-NF-κB, and n-NF-κB. β-actin was served as whole cell loading control and Lamin A was used as nuclear loading control. Relative expression was quantified by ImageJ. (**B**) The effect of MSA (2 μM, 6 h) on the nuclear translocation of NF-κB was detected by immunofluorescence. (**C**) Relative mRNA expressions of NF-κB downstream moleculars (ICAM-1 and VCAM-1) were quantified by qPCR assay after MSA treatment (2 μM, 0, 3, 6, 12 h). A 2^−ΔΔCT^ relative quantification method was employed and β-actin was used as housekeeping gene. The data in Fig. 7 were determined by one way ANOVA comparison test and the *error bars* represented the SD (n = 3). Different letters indicate statistically significant differences between groups (*p* < 0.05).

The phosphorylation and nuclear translocation of NF-κB are downstream steps resulting from integrin activation and AKT phosphorylation. Hence, western blot was further employed to investigate the effect of MSA on NF-κB. MSA not only inhibited the phosphorylation of IκBα (25.1%) and NF-κB (8.29%) within a short time, but also down-regulated the nuclear localization of NF-κB (37.39%, Fig. 7A). Immunofluorescence was used to further evaluate the effect of MSA on the nuclear translocation of NF-κB. MSA treatment led to relatively lower fluorescence intensity (60.93%) and no nuclear NF-κB were observed (Fig. 7B). Additionally, we performed qPCR to measure the mRNA levels of ICAM-1 and VCAM-1, which are both target genes of NF-κB. After exposure to MSA, the mRNA levels of ICAM-1 (3.00%) and VCAM-1 (4.75%) were down-regulated within a short period (Fig. 7C). To evidence the relationship between integrin β3 downregulation induced by MSA and the sequential inhibition of AKT/NF-κB phosphorylation and angiogenesis, pcDNA3.1-beta-3^29^ or β3-siRNA was transfected into HUVECs to induce overexpression or knockdown of integrin β3, and treated with or without MSA in the meanwhile. As shown in the Supplementary Fig. S4, all of the groups treated with MSA displayed obvious decreases of integrin β3 and resulted in the subsequent changes of AKT and NF-κB phosphorylation, whereas pcDNA3.1-beta-3 induced transient high expression of integrin β3 and rescued those effects to some extent. In consistent with the integrin β3 signaling, promotion of cells adhesion, suppression of cells migration and tubes formation were shown in the Supplementary Fig. S5, in which also showed a compensation by overexpression of integrin β3. Together, these results preliminarily elucidated the mechanism of inhibition angiogenesis induced by MSA through downregulation of integrin β3 signaling and AKT and NF-κB phosphorylation.

## Discussion

A major goal of cancer biologists over the past few decades has been to develop anti-tumor drugs that limit angiogenesis and thereby reduce oxygen and nutrient supplies required for tumor growth^1, 2^. Given the potential side effects and high costs associated with these compounds, interest has emerged in focusing on natural compounds because of their biocompatibility. As a metabolite of Se from animal cells, MSA offers potential due to its widely reported anti-tumor effects at super nutritional levels^20, 24, 26, 30^. However, little information is available about MSA regarding anti-angiogenic effects and mechanisms of its actions. In this study, we present evidence that MSA at nutritional Se levels could inhibit VEGF-induced tube formation *in vitro*, and neo-angiogenesis *ex vivo* and *in vivo* without inducing apoptosis. Moreover, MSA could inhibit angiogenesis by both down-regulating the expression and disrupting the clustering of integrin β3, which further inhibit the phosphorylation of AKT, IκBα, and NF-κB, as well as the nuclear translocation of NF-κB.

Generally, the narrow margin between effective and toxic doses of Se as well as its metabolites like MSA, makes it difficult to identify optimal dosages lacking cytotoxicity to normal cells. According to a previous study^23^, Se concentrations in blood ranging from 0.71 to 3.24 μM are considered in the nutritional range. As shown in our results, addition of 2 µM MSA increased the Se concentration in media by 153.03 ng/mL (1.94 μM), which falls within nutritional Se concentrations. Meanwhile, the endogenous Se levels including total Se and free Se in FBS, medium, cultured supernatant and HUVECs were around 1 μM or less, and showed no obvious changes of angiogenesis, which indicated that their effects on angiogenesis might not be dominate in our experiment conditions. Moreover, HPLC-ICP-MS results showed that SeMC, SeMet, and DMSe seemed to be the major Se species in HUVECs exposing to MSA. However, the peak of MSA was failed to be detected in our experiment conditions, it was probably because the dosage of MSA we used were relatively lower and MSA could be converted to other species rapidly even after a short time exposure according to the previous work^31^.

Moreover, an MTT assay showed that doses above 5 µM had obvious inhibitory effects on cell proliferation, while the cytotoxicity of MSA was relatively lower compared with other Se compounds. Together, with the results of Rodamine-123 staining, PI staining, and western blot, these results suggested that MSA within nutritional Se concentrations had no effect on cell proliferation or induce apoptosis even in the presence of VEGF, but still could inhibit angiogenesis *in vitro*, *ex vivo* and *in vivo*.

Although the *ex vivo* and *in vivo* experiments beyond 48 h, culture medium supplemented with MSA or VEGF were changed every other days during two weeks in aortic ring assay and the CAM assay was just performed within 72 h. Moreover, considering not only the inhibition effects of MSA on cell migration and tube formation which are crucial for initiation of angiogenesis during the early 12–24 h, but also the downregulation effects of MSA on the protein level of integrin β3 and phosphorylated AKT within 24 h, thus supplementary MSA could maintain its activity during the whole procedure *ex vivo* or *in vivo* which was consistent with the dynamic alteration of Se concentration in cultures.

The family of integrins consists of eighteen α and eight β subunits, which form more than two dozen unique integrin heterodimers that are widely expressed in human tissues including endothelial cells^8^. There are considerable data suggesting that integrin β3 is prominently expressed in endothelial cells and cancer cells^32^. Integrin β3 is a crucial connective molecular for recognizing proteins containing Arg-Gly-Asp (RGD) sequence^33^, which relates to its regulation of cell attachment, migration, and outside-in pathways during angiogenesis. Our qPCR results showed that MSA could down-regulate integrin αv, β1, and β3 at the level of mRNA. However, at the level of protein, only integrin β3 was down-regulated in a time- and dose-dependent manner. Moreover, it is reported that motile cells exhibit functional polarity with a large lamellipodia at the leading edge^34^, where integrin β3 exhibit a polar surface distribution^9, 10^ and participate in forming complexes with RPTPα for rigidity sensing^35^. Our data showed that integrin β3 clustered at the surface of endothelial cells exposed to VEGF, and this process could be disrupted by MSA. Together, these findings suggested that MSA could inhibit cell migration by down-regulating and disrupting the clustering of integrin β3.

After activation, integrins transduce outside-in signal by phosphorylation of FAK, which is a key initiator of focal adhesion complex formation. The phosphorylation cascade of FAK, PI3K, AKT, IKKα, and IκBα is a crucial pathway downstream of integrin activation. Phosphorylation and nuclear translocation of NFκB are mechanisms by which several genes are actively transcribed^36–38^. Our results show that MSA inhibits the phosphorylation of AKT and IκBα, which subsequently inhibits the phosphorylation and nuclear localization of NFκB. The mRNA expression of NFκB responsive genes, like ICAM-1 and VCAM-1, were also found to be down-regulated.

In addition, to clarify the relationship between integrin β3 downregulation induced by MSA and the sequential inhibition on AKT/NF-κB phosphorylation and angiogenesis, overexpression or knockdown of integrin β3 were employed individually. As shown in our results, downregulation of integrin β3 could be observed in cells exposed to MSA. However, transfection with pcDNA3.1-beta-3 could obviously induced transient upexpression of integrin β3, which could rescued the downregulation of integrin β3 induced by MSA as well as the subsequent inhibitory effects of MSA on AKT and NF-κB phosphorylation or angiogenesis in some degree. Together, these results suggested that integrin β3 downregulation by MSA could sequentially inhibit AKT/NF-κB phosphorylation and angiogenesis.

Overall, our results suggest that MSA within nutritional Se concentrations effectively inhibited angiogenesis *in vitro*, *ex vivo* and *in vivo* by down-regulating integrin 3β levels and disrupting its clustering. MSA treatment led to an inhibition of the phosphorylation of AKT and IκBα, which further inhibited the phosphorylation and nuclear translocation of NFκB. Our results demonstrated that MSA may represent a potent therapeutic agent for anti-cancer by inhibiting tumor angiogenesis in clinical applications.

## Materials and Methods

### Cell lines, cell culture, and reagents

MSA (CH_3_SeO_2_H) was purchased from Sigma. A 2 mM stock solution of MSA was prepared in PBS, stored at −20 °C, and then diluted to final required concentrations in cell culture medium.

HUVECs were obtained from Sun Yat-Sen University. HUVECs were cultured in Dulbecco’s Modified Eagle Medium: Nutrient Mixture F-12 Medium (ThermoFisher, Gibco) supplemented with 10% Fetal Bovine Serum (FBS, Life Technology, Gibco), 100 U/mL penicillin, and 0.1 mg/ml streptomycin at 37 °C in a humidified incubator with 5% CO_2_ atmosphere.

### Atomic fluorescence spectrometer

HUVECs (2 × 10^5^ per well) were seeded in 6-well plates and treated with 2 µM MSA for different hours (0, 0.5, 6, 12, 24, 48 h) in a reverse time-course method. Media and cells were collected. After detecting the concentration of protein, samples were digested with mix acid (nitric acid: perchloric acid = 4:1), terminated with 6 mol/L hydrochloric acid, and diluted with 10% hydrochloric. The Se concentrations of samples were detected by AFS (AF-2200a, Beifeng-Ruili).

### ICP-MS

HUVECs (2 × 10^5^ per well) were seeded in 6-well plates and treated with 2 µM MSA for different hours (0, 0.5, 6, 12, 24 h). Different Se standards including MSA, DL-selenomethionine (Se-Met), Se-L-methylselenocysteine (Se-MC), and DMSe were used as positive controls, cells treated without MSA were served as negative control. Cell lysates were digested by using the MARS-5 microwave system (CEM). Quantification of specific Se species in cell lysates were performed by HPLC for chromatographic separations on an Agilent Zorbax Rx-C8 column coupled directly to collision reaction cell ICP-MS (Agilent 7500ce, Palo Alto) according to our previous method^39^. Calibration was performed by the standard addition technique using peak area measurements of the chromatographic signals by monitoring the isotopes ^78^Se and ^77^Se.

### Cell proliferation assay

HUVECs (8 × 10^3^ per well) were treated with different concentrations (0–10 µM) of MSA or other Se compounds (SeMet, Selenomethionine; SeCys, selenocysteine; and Se (IV), sodium selenite) for 24 h. Cell proliferation was detected by MTT (Sigma) assay with Microplate Reader (Molecular Devices).

### Rhodamine-123 and Propidium Iodide (PI) staining

HUVECs (4 × 10^5^ per well) were seeded in 6-well plates and treated with different concentrations of MSA or 80 µM cisplatin for 24 h. Cells were collected and stained with 200 mM Rhodimine-123 (Sigma) at 37 °C in dark for 20 min. After washing with PBS, cells were evaluated for fluorescence by a flow cytometer.

For PI staining, treated cells were collected and fixed with pre-cooled 70% ethanol at −20 °C overnight. After washing with PBS and 1% BSA in PBS, cells were stained with 10 μg/mL PI (Sigma) overnight at 4 °C in dark. After staining, cells were detected by flow cytometer (Beckman Coulter, Gallios).

### Cell adhesion and Migration assays

Adhesion assays were performed as described by Chen and colleagues^40^. In brief, 96-well plates were pre-coated with 1 mg/ml Collagen I (Coring) at 37 °C for 1 h. After treating with 2 µM MSA or 50 ng/ml VEGF (Peprotech) for 24 h, cells (2 × 10^4^ per well) were reseeded in 96-well plates. Cells were allowed to adhere for 20 min, then non-adherent cells were washed away. Adherent cells were cultured for another 4 h and quantified following a MTT assay protocol described above.

For migration analyses, HUVECs (2 × 10^5^ per well) were seeded in 6-well plates and serum starved. Cells were wounded by 200 µL pipette tips, washed, and incubated with 50 ng/mL VEGF or 2 µM MSA. Images were taken after 24 h. The migration distances were quantified by using the software Adobe Photoshop CS6.

### Assays measuring Tube formation, Aortic rings, and CAM

To measure tube formation, HUVECs (2 × 10^4^ per well) were seeded in 48-well plates pre-coated with Matrigel (Corning), starved, and treated with 50 ng/ml VEGF or 2 µM MSA. Images were taken after 6 h. The tube lengths were quantified by using the software Adobe Photoshop CS6.

Aortic ring assays were performed as previously described with minor modifications^41^. Aortas were isolated from 6-week-old C57BL/6 mice, cleaned off the fatty tissue, and cut into 1-mm-long rings. After washing, the rings were serum starved with Opti-MEM (Life Technology, Gibco) for 18 h. Then, one aortic ring was embedded per well of a 96-well plate with 1 mg/mL collagen at 37 °C for 1 h, and then 150 µL Opti-MEM medium supplemented with 2% FBS and 50 ng/ml VEGF or 2 µM MSA was added. Medium were refreshed every other day and images were taken after 14 d.

For CAM assays, groups of 6 fertilized chicken eggs were incubated at 37.5 °C in an incubator with 60% humidity. After incubating for 7 d, eggs were punched, injected with 200 µL PBS or 2 µM MSA, and incubated for another 3 d. The CAMs were then imaged. A quantitative analysis of the vascular network was performed as previously described with some modification^42^. In brief, images were converted into gray-scale and analyzed using software ImageJ. The algorithm input parameters were initially set to obtain the identification of pixels related to the blood vessels as strong and positive as to the background and weak positive and tuned to minimize non-specific pixel recognition as strong positive. The ratio between the number of strong positive pixels and the sum of weak, background and strong positive pixels is the morphometric value used to quantified the blood vessels density across the experimental groups. All the analysis were performed on images with equal area. The CAMs were collected, weighed and digested as described above and Se concentrations were detected by AFS.

### Immunofluorescence

HUVECs (2 × 10^4^ per well) were seeded onto coverslips (NEST, d = 16 mm) pre-coated with poly-L-lysine (Sigma, MW = 70000–150000), and treated with VEGF or MSA for 24 h. Coverslips were fixed with 4% paraformaldehyde, and then blocked with 10% goat serum. Cells were then incubated with anti-integrin β3 antibody (Abcam, ab34409, 1:100) at 4 °C overnight and Cy3-conjuated goat anti-mouse IgG (EarthOx, E031620, 1:200) at room temperature for 1 h. Nuclei were stained with DAPI (Sigma, 0.1 mg/mL) for 15 min. After sealing, coverslips were photographed by confocal laser scanning microscope (Olympus, FV10i).

### Real-time PCR analysis and Western blotting

HUVECs (1.6 × 10^5^ per well) were treated with 2 µM MSA for different periods (0–12 h). Total RNA was isolated with RNAPrep pure Cell/Bacterial Kit (TianGen), and 1 µg aliquots of the isolated total RNA were reverse transcribed with FASTQuant RT Kit (TianGen). The mRNA levels of integrin subunits αv, α1,α5, β1, β3, β5 were further quantified by using TransStart Tip Green qPCR Supermix (TransGen Biotech) and Bio-Rad CFX96 system. Levels of β-actin mRNA were used as housekeeping transcript. The amounts of target gene were estimated by 2^−ΔΔCT^ relative quantification method. The primers for qPCR were provided in supplementary information.

RIPA (Life Technology, Invitrogen) containing protease inhibitor cocktail (Sigma) was used to lyse cell samples. The insoluble fraction was removed by centrifugation at 10000 × g for 10 min at 4 °C. The concentration of total protein in supernatant was measured by BCA assay (Pierce, Life Technology). After adjusting to equal concentration with loading buffer (Bio-Rad), protein samples were boiled, separated by SDS-PAGE, and transferred to a PVDF membrane (0.22 µm, Millipore). Membranes were blotted by 5% nonfat milk (BD) in TBST for 1 h. The membranes were then incubated with primary antibodies (Cell Signaling, 1:1000) at 4 °C overnight, and HRP-conjugated secondary antibody (Cell signaling, 1:3000) for 1 h at room temperature. Protein bands were visualized on X-ray film (Kodak) by using ECL Assay Kits (Pierce, Life Technology). The relative levels of protein were quantified according to the reference band of β-actin.

### Statistical analysis

Experiments were carried out at least in triplicate. The data were presented as mean ± SD. Statistical analysis was performed using SPSS 20.0 and statistical comparisons between groups were performed by different analysis which described in figure legend. *P* value of <0.01 or <0.05 was considered statistically significant, while different letters indicate statistically significant differences between groups (*p* < 0.05).

### Ethical approval

All animal experiments in this study were approved by the Animal Care and Ethics Committee of Jinan University (20150306003). Principles of laboratory animal care were followed and all procedures were conducted according to the guidelines established by the National Laboratory Animal Ethics Committee of China, and every effort was made to minimize suffering.

## Electronic supplementary material

Supplementary information for Methylseleninic Acid Provided at Nutritional Selenium Levels Inhibits Angiogenesis by Down-regulating Integrin β3 Signaling

## References

[1] Chung AS, Lee J, Ferrara N (2010). Targeting the tumour vasculature: insights from physiological angiogenesis. Nature reviews. Cancer.

[2] Ferrara N, Adamis AP (2016). Ten years of anti-vascular endothelial growth factor therapy. Nature reviews. Drug discovery.

[3] Pang X (2009). Morelloflavone, a biflavonoid, inhibits tumor angiogenesis by targeting rho GTPases and extracellular signal-regulated kinase signaling pathways. Cancer research.

[4] Kalluri R (2003). Basement membranes: structure, assembly and role in tumour angiogenesis. Nature reviews. Cancer.

[5] Szekanecz Z, Koch AE (2007). Mechanisms of Disease: angiogenesis in inflammatory diseases. Nature clinical practice. Rheumatology.

[6] Ferrara N, Gerber HP, LeCouter J (2003). The biology of VEGF and its receptors. Nature medicine.

[7] Goel HL, Mercurio AM (2013). VEGF targets the tumour cell. Nature reviews. Cancer.

[8] Foubert P, Varner JA (2012). Integrins in tumor angiogenesis and lymphangiogenesis. Methods in molecular biology (Clifton, N.J.).

[9] Hood JD, Cheresh DA (2002). Role of integrins in cell invasion and migration. Nature reviews. Cancer.

[10] Mayor R, Etienne-Manneville S (2016). The front and rear of collective cell migration. Nature reviews. Molecular cell biology.

[11] Shattil SJ, Kim C, Ginsberg MH (2010). The final steps of integrin activation: the end game. Nature reviews. Molecular cell biology.

[12] Robinson SD, Hodivala-Dilke KM (2011). The role of beta3-integrins in tumor angiogenesis: context is everything. Current opinion in cell biology.

[13] Collins T (1995). Transcriptional regulation of endothelial cell adhesion molecules: NF-kappa B and cytokine-inducible enhancers. FASEB journal: official publication of the Federation of American Societies for Experimental Biology.

[14] Shih RH, Wang CY, Yang CM (2015). NF-kappaB Signaling Pathways in Neurological Inflammation: A Mini Review. Frontiers in molecular neuroscience.

[15] Marcu KB, Otero M, Olivotto E, Borzi RM, Goldring MB (2010). NF-kappaB signaling: multiple angles to target OA. Current drug targets.

[16] Ahn KS, Aggarwal BB (2005). Transcription factor NF-kappaB: a sensor for smoke and stress signals. Annals of the New York Academy of Sciences.

[17] Willett M, Pollard HJ, Vlasak M, Morley SJ (2010). Localization of ribosomes and translation initiation factors to talin/beta3-integrin-enriched adhesion complexes in spreading and migrating mammalian cells. Biology of the cell / under the auspices of the European Cell Biology Organization.

[18] Zaidel-Bar R, Ballestrem C, Kam Z, Geiger B (2003). Early molecular events in the assembly of matrix adhesions at the leading edge of migrating cells. Journal of cell science.

[19] Schomburg L (2012). Selenium, selenoproteins and the thyroid gland: interactions in health and disease. Nature reviews. Endocrinology.

[20] Zhang Y (2014). Synergistic induction of apoptosis by methylseleninic acid and cisplatin, the role of ROS-ERK/AKT-p53 pathway. Molecular pharmaceutics.

[21] Cai X (2016). Selenium Exposure and Cancer Risk: an Updated Meta-analysis and Meta-regression. Scientific reports.

[22] Levander OA (1991). Scientific rationale for the 1989 recommended dietary allowance for selenium. Journal of the American Dietetic Association.

[23] Diplock AT (1993). Indexes of selenium status in human populations. American Journal of Clinical Nutrition.

[24] Tarrado-Castellarnau M (2015). Methylseleninic acid promotes antitumour effects via nuclear FOXO3a translocation through Akt inhibition. Pharmacological research.

[25] Sinha I, Allen JE, Pinto JT, Sinha R (2014). Methylseleninic acid elevates REDD1 and inhibits prostate cancer cell growth despite AKT activation and mTOR dysregulation in hypoxia. Cancer medicine.

[26] Wang L (2016). Methylseleninic Acid Superactivates p53-Senescence Cancer Progression Barrier in Prostate Lesions of Pten-Knockout Mouse. Cancer prevention research (Philadelphia, Pa.).

[27] Suzuki KT, Tsuji Y, Ohta Y, Suzuki N (2008). Preferential organ distribution of methylselenol source Se-methylselenocysteine relative to methylseleninic acid. Toxicology and applied pharmacology.

[28] Goenaga-Infante H, Kassam S, Stokes E, Hopley C, Joel SP (2011). Capabilities of HPLC with APEX-Q nebulisation ICP-MS and ESI MS/MS to compare selenium uptake and speciation of non-malignant with different B cell lymphoma lines. Analytical and bioanalytical chemistry.

[29] Takagi J, Petre BM, Walz T, Springer TA (2002). Global conformational rearrangements in integrin extracellular domains in outside-in and inside-out signaling. Cell.

[30] Qi Y (2012). Methylseleninic acid enhances paclitaxel efficacy for the treatment of triple-negative breast cancer. PloS one.

[31] Jüliger S, Goenaga-Infante H, Lister TA, Fitzgibbon J, Joel SP (2007). Chemosensitization of B-Cell Lymphomas by Methylseleninic Acid Involves Nuclear Factor-κB Inhibition and the Rapid Generation of Other Selenium Species. Cancer Research.

[32] Plow EF, Meller J, Byzova TV (2014). Integrin function in vascular biology: a view from 2013. Current opinion in hematology.

[33] Atkinson SJ, Ellison TS, Steri V, Gould E, Robinson SD (2014). Redefining the role(s) of endothelial alphavbeta3-integrin in angiogenesis. Biochemical Society transactions.

[34] Petrie RJ, Yamada KM (2012). At the leading edge of three-dimensional cell migration. Journal of cell science.

[35] von Wichert G (2003). RPTP-alpha acts as a transducer of mechanical force on alphav/beta3-integrin-cytoskeleton linkages. The Journal of cell biology.

[36] Perkins ND (2012). The diverse and complex roles of NF-kappaB subunits in cancer. Nature reviews. Cancer.

[37] Oeckinghaus A, Hayden MS, Ghosh S (2011). Crosstalk in NF-kappaB signaling pathways. Nature immunology.

[38] Hayden MS, Ghosh S (2011). NF-kappaB in immunobiology. Cell research.

[39] Huang Z (2008). Low selenium status affects arsenic metabolites in an arsenic exposed population with skin lesions. Clinica chimica acta; international journal of clinical chemistry.

[40] Chen Y (2009). Combined integrin phosphoproteomic analyses and small interfering RNA–based functional screening identify key regulators for cancer cell adhesion and migration. Cancer research.

[41] Baker M (2012). Use of the mouse aortic ring assay to study angiogenesis. Nature protocols.

[42] Magnaudeix A (2016). Quantitative analysis of vascular colonisation and angio-conduction in porous silicon-substituted hydroxyapatite with various pore shapes in a chick chorioallantoic membrane (CAM) model. Acta biomaterialia.

