# Author Correction: Methylseleninic Acid Provided at Nutritional Selenium Levels Inhibits Angiogenesis by Down-regulating Integrin β3 Signaling

**DOI:** 10.1038/s41598-020-62438-5

**Published:** 2020-03-24

**Authors:** Zhihui Cai, Liangbo Dong, Chengwei Song, Yanqing Zhang, Chenghui Zhu, Yibo Zhang, Qinjie Ling, Peter R. Hoffmann, Jun Li, Zhi Huang, Wei Li

**Affiliations:** 1 Department of Biotechnology, Jinan University, Guangzhou, Guangdong Province China; 2 College of Pharmacy, Jinan University, Guangzhou, Guangdong Province China; 3 Department of Cell and Molecular Biology, John A. Burns School of Medicine, University of Hawaii, Honolulu, Hawaii USA

Correction to: *Scientific Reports* 10.1038/s41598-017-09568-5, published online 25 August 2017

This Article contains errors.

Figure 3A is mislabelled and the corresponding figure caption is incorrect. The correct version of Figure 3 appears below as Figure 1.

**Figure 1:**
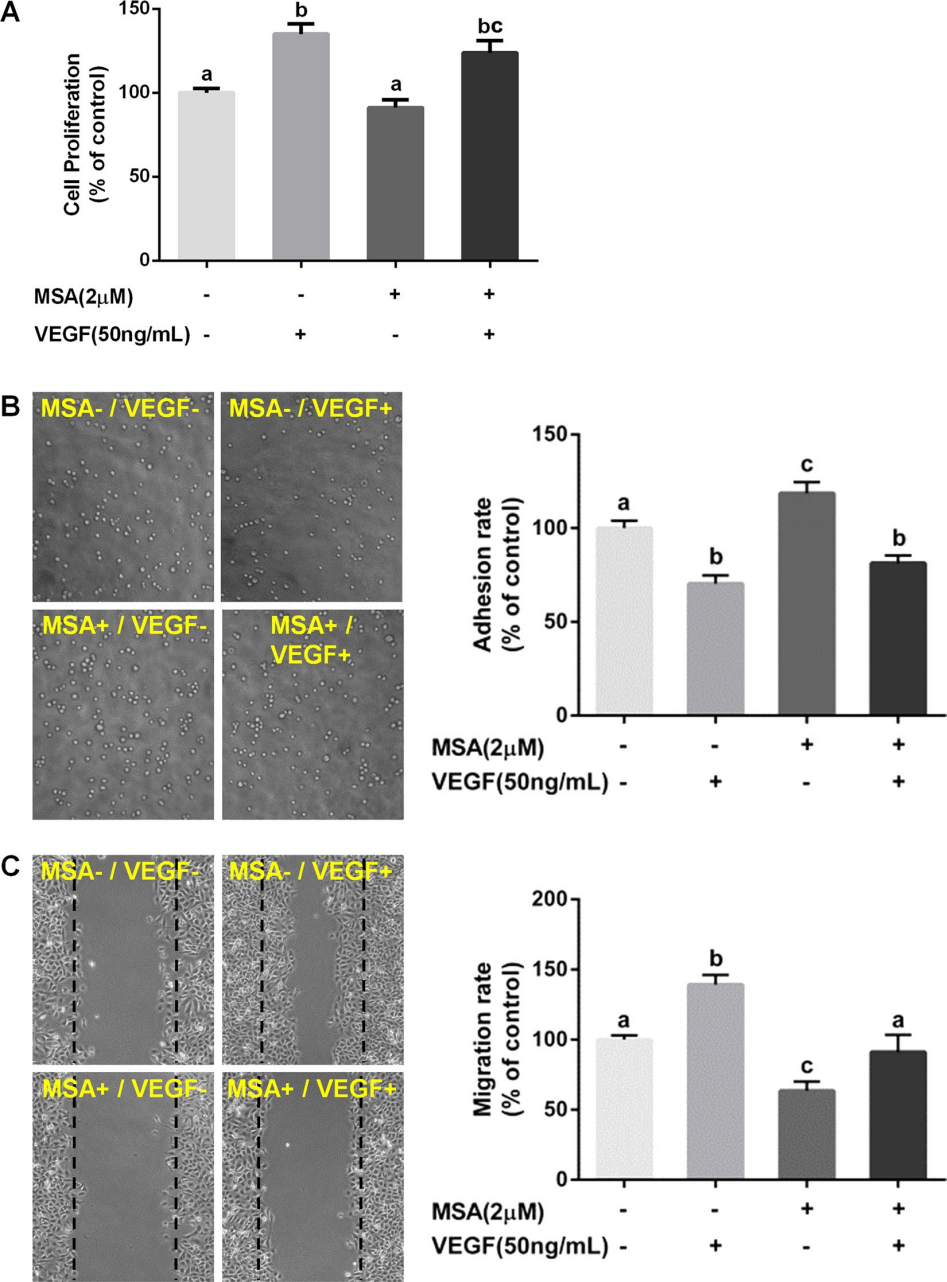
MSA increases cell adhesion but inhibits cell migration. HUVECs were treated with MSA (2 μM) and/or VEGF (50 ng/mL) for 24 h. (**A**) Cell proliferation and (**B**) numbers of adherent cells were quantified by MTT assay. (**C**) The healing distances were measured by Photoshop CS6 software. The data in Fig 3 were determined by one way ANOVA comparison test and the error bars represented the SD (n = 3). Different lowercase letters (a-c, p < 0.01) above the bars to show the statistically significant differences between groups.

