# Supplementary material for: Methylseleninic Acid Provided at Nutritional Selenium Levels Inhibits Angiogenesis by Down-regulating Integrin β3 Signaling
Source: Sci Rep. 2017 Aug 25;7:9445. doi: 10.1038/s41598-017-09568-5 (PMC5573405; doi:10.1038/s41598-017-09568-5)
Supplement: Supplementary file 1 — Supplementary information for Methylseleninic Acid Provided at Nutritional Selenium Levels Inhibits Angiogenesis by Down-regulating Integrin β3 Signaling [file 41598_2017_9568_MOESM1_ESM.pdf]

Supplementary Information

Methylseleninic Acid Provided at Nutritional Selenium Levels Inhibits Angiogenesis by Down-regulating Integrin β3 Signaling

Zhihui Cai[1], Liangbo Dong[1], Chengwei Song[1], Yanqing Zhang[1], Chenghui Zhu[1,2], Yibo Zhang[1], Qinjie Ling[1], Peter R. Hoffmann[3], Jun Li[1], Zhi Huang[1]*, Wei Li[1]*

[1] Department of Biotechnology, Jinan University, Guangzhou, Guangdong Province, China

[2] College of Pharmacy, Jinan University, Guangzhou, Guangdong Province, China

[3] Department of Cell and Molecular Biology, John A. Burns School of Medicine, University of Hawaii, Honolulu, Hawaii U.S.A

***Correspondence:** Zhi Huang and Wei Li

E-mail: thsh@jnu.edu.cn

Supplementary Materials and Methods

Total Se and free Se preparation

Total Se from FBS, complete DMEM/F-12 medium, supernatant and HUVECs were collected and in which free Se (non-protein binding) were separated by centrifugation through a Microsep Advance centrifugal devices (PALL,1kDa) at 7500 ×g for 30 min at 4℃. Free Se was added as supplement in DMEM/F-12 medium for further study. The Se levels of total Se and free Se were detected by AFS. The effects of endogenous Se on cell adhesion, migration and tube formation were performed as described above in materials and methods.

Plasmids, siRNAs, and transfection

The pcDNA3.1-beta-3 was purchased from Addgene (Plasmid 27289) and pcDNA3.1(+)/myc-His A was purchased from Invitrogen (ThermoFisher, V80020). Integrin β3 siRNA (h, sc-29375) and negative control (NC) siRNA (sc-37007) were purchased from Santa Cruz.

For transfection, HUVECs (2×10^5 per well) were seeded in 6-well plates in 2 mL complete growth medium. After 24 h, for each well of cells to be transfected, 2.5 μg of plasmid DNA was transfect with Lipofectamine LTX and PLUS Reagent (Life Technology) or 25 pmol of siRNA was transfect with Lipofectamine RNAiMAX (Life Technology) following the protocol and cells were treated with or without MSA in the meanwhile. After another 24-hours-incubation, cells were used for different assay.

Supplementary Table S1.

Sequence of primers for qPCR.

Gene		Primer	Product (bp)	Accession number
ITAV	Forward	GGCTGCATATTTCGGATTTTCTG	183	NM_001145000.2
	Reverse	CCATTCAGCTTTGTCGTCTGG		
ITA1	Forward	GTGCTTATTGGTTCTCCGTTAGT	208	NM_181501.1
	Reverse	CACAAGCCAGAAATCCTCCAT		
ITA5	Forward	AGACATTCGATCCCTCTACAACT	249	NM_002205.4
	Reverse	AATCGGCCAAACTCATCATGG		
ITB1	Forward	GTAACCAACCGTAGCAAAGGA	98	NM_002211.3
	Reverse	TCCCCTGATCTTAATCGCAAAAC		
ITB3	Forward	AGTAACCTGCGGATTGGCTTC	164	NM_000212.2
	Reverse	GTCACCTGGTCAGTTAGCGT		
ITB5	Forward	GGAAGTTCGGAAACAGAGGGT	106	NM_002213.4
	Reverse	CTTTCGCCAGCCAATCTTCTC		
ACTIN	Forward	GTGCTATGTTGCTCTAGACTTCG	174	NM_001101.3
	Reverse	ATGCCACAGGATTCCATACC		
ICAM1	Forward	TTGGGCATAGAGACCCCGTT	82	NM_000201.2
	Reverse	GCACATTGCTCAGTTCATACACC		
VCAM1	Forward	TTTGACAGGCTGGAGATAGACT	173	NM_001199834.1
	Reverse	TCAATGTGTAATTTAGCTCGGCA		

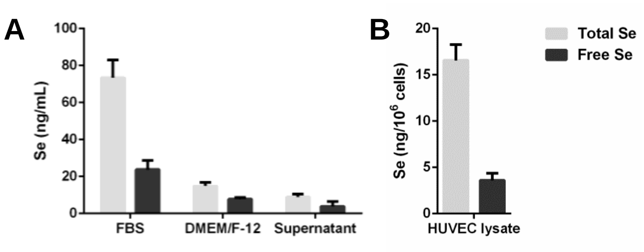

Supplementary Fig S1: Detection of endogenous Se levels. Total Se from FBS, complete DMEM/F-12 medium, supernatant and HUVECs were collected directly and in which free Se were separated by ultrafiltration (1kDa cut off). The concentrations of total Se and free Se were detected by AFS.

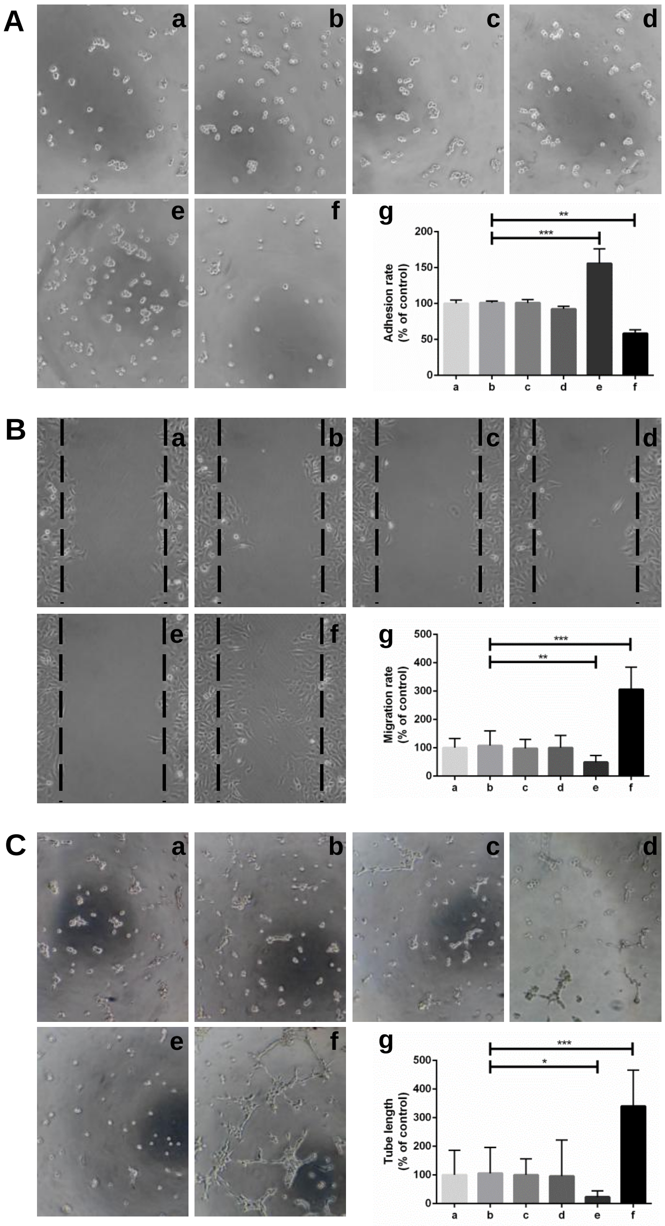

Supplementary Fig S2: Effects of endogenous MSA/Se on angiogenesis *in vitro*. HUVECs were cultured in medium (a), medium supplement with FBS (b), medium supplement with ultrafitration of FBS (c), ultrafitration of HUVECs medium (d), or medium supplement with MSA (e), or medium supplement with VEGF (f). Cell adhesion **(A)**, cell migration**(B),** and tube formation **(C)** were detected and quantified individually. The data were determined by nonparametric tests and the *error bars* represented the SD (A-B: n=3; C: n=60). *$p < 0.05$, **$p < 0.01$, ***$p < 0.001$.

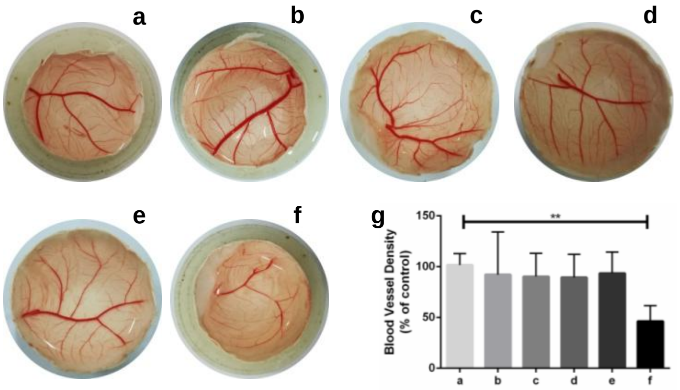

Supplementary Fig S3: Effects of endogenous MSA/Se on angiogenesis *in vivo*. Fertilized chicken eggs were injected with 200 μL PBS (a), DMEM/F-12 medium (b), ultrafitration of medium (c), FBS (d), ultrafitration of FBS (e), or medium supplement with MSA (f) and incubated for 3 d. The areas of vessels on CAM were quantified by Adobe Photoshop CS6. The *error bars* represented the SD (n=6, $^{**}p < 0.01$) and were determined by nonparametric tests.

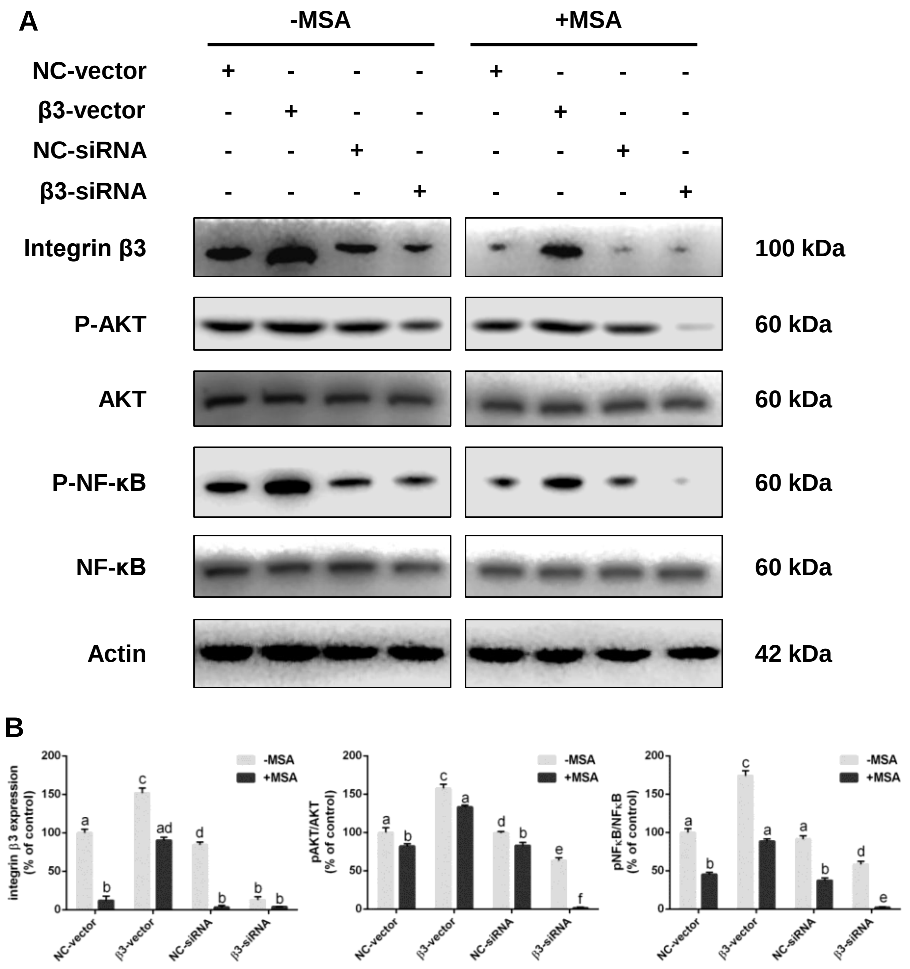

Supplementary Fig S4: Effects of MSA on phosphorylation of AKT and NF-κB under integrin β3 upregulation or silencing. HUVECs were transfected with pcDNA3.1-beta-3 (β3-vector), pcDNA3.1(+)/myc-His (NC-vector), NC-siRNA or β3-siRNA. After co-treated with/without MSA for 24 h in the meanwhile, western blot **(A)** was employed to analyze the protein levels β3 and the phosphorylation of AKT and NF-κB and β-actin was served as loading control. The relative levels of protein were quantified by ImageJ software. The error bars **(B)** represented the SD (n=3) and were determined by two-way ANOVA comparison test. Different letters indicate statistically significant differences between groups ($p < 0.05$).

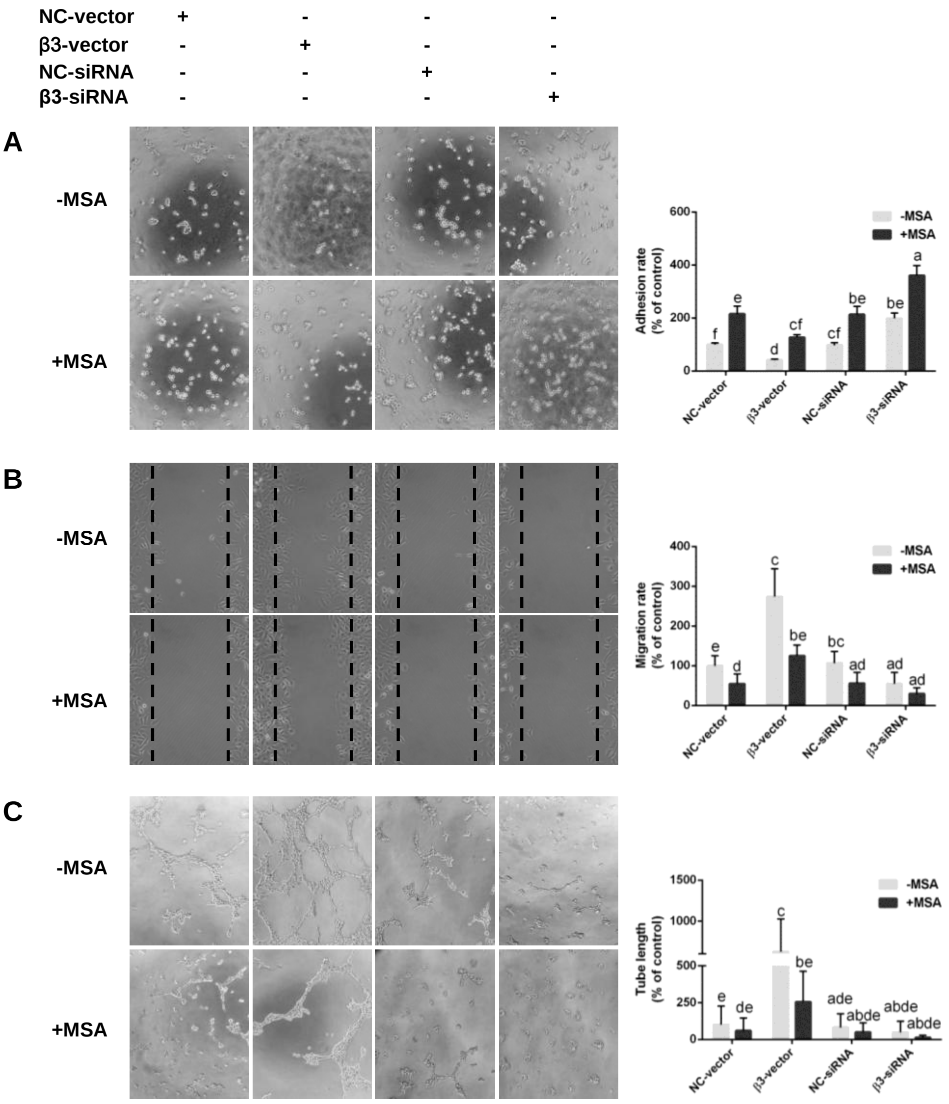

Supplementary Fig S5: Effects of MSA on angiogenesis under integrin β3 upregulation or silencing. HUVECs were transfected with pcDNA3.1-beta-3 (β3-vector), pcDNA3.1(+)/myc-His (NC-vector), NC-siRNA or β3-siRNA. After co-treated with/without MSA for 24 h in the meanwhile, cell adhesion **(A)**, cell migration **(B)**, and tube formation **(C)** were detected and quantified individually. Data were determined by two-way ANOVA comparison test and the *error bars* represented the SD (A-B: n=3; C: n=60). Different letters indicate statistically significant differences between groups ($p < 0.05$).

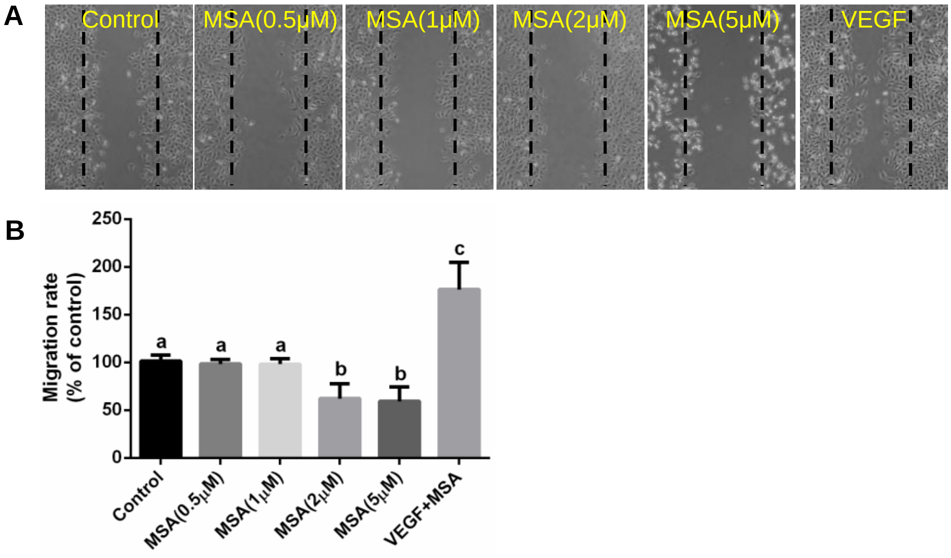

Supplementary Fig S6: Dose dependency of MSA on cell migration. HUVECs were treated with MSA (0, 0.5, 1, 2, 5 μM) or VEGF. Images **(A)** were captured after healing for 24 h. **(B)** The healing distances were measured by Photoshop CS6 software. The data were determined by one-way ANOVA comparison test and the error bars represented the SD (n=3). Different letters indicate statistically significant differences between groups ($p < 0.05$).

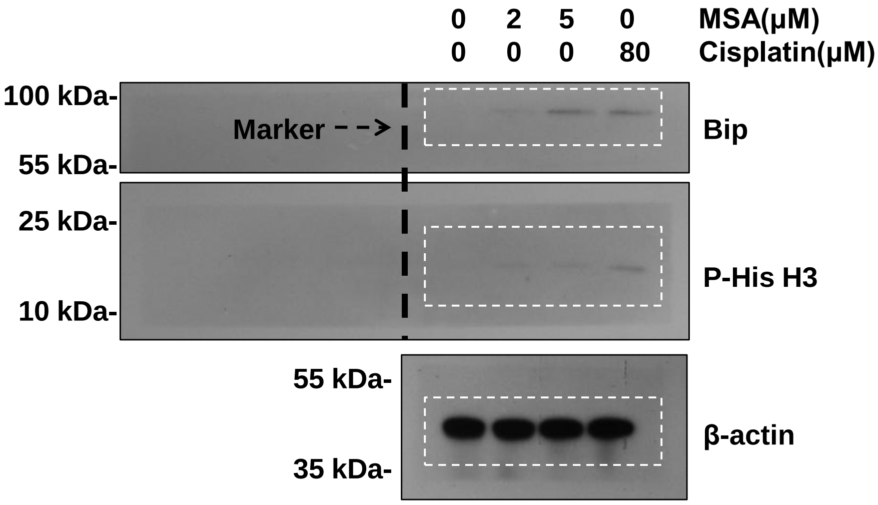

Supplementary Fig S7: Original Western Blot of Figure 2E.

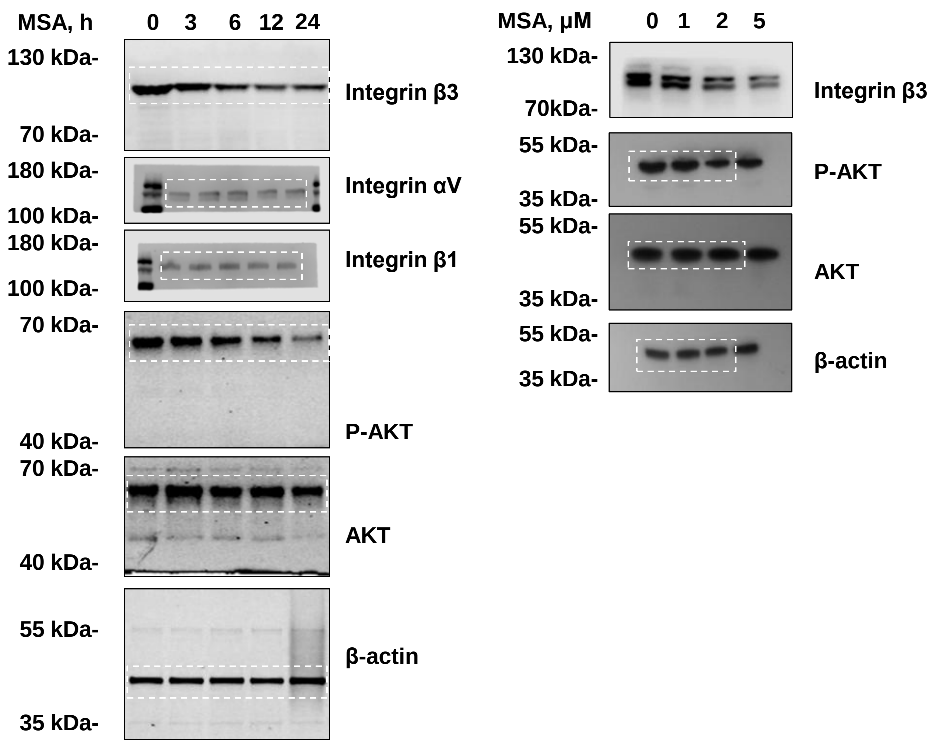

Supplementary Fig S8: Original Western Blot of Figure 4B and 4C.

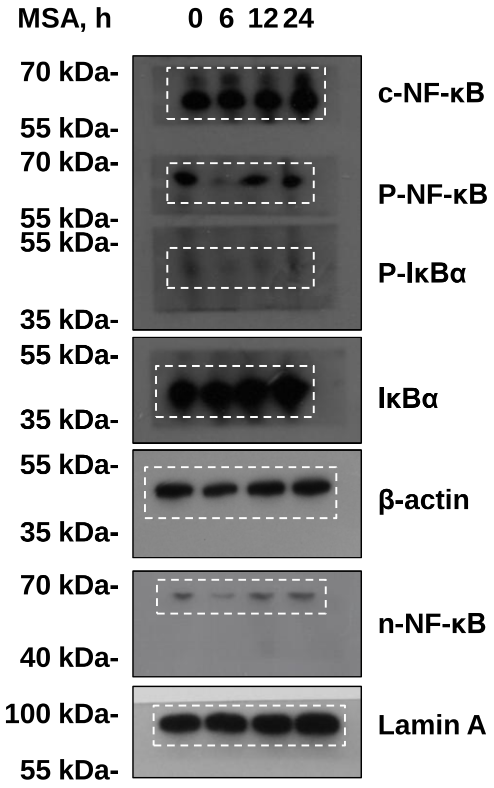

Supplementary Fig S9: Original Western Blot of Figure 7A.
